# ﻿On four new species of the orb-weaver spider genus *Araneus* Clerck, 1757 (Araneae, Araneidae) from southern China

**DOI:** 10.3897/zookeys.1160.101594

**Published:** 2023-05-09

**Authors:** Yibei Wu, Cheng Wang, Nanfei Wu, Mengfei Zhang, Xiaoqi Mi

**Affiliations:** 1 College of Agriculture and Forestry Engineering and Planning, Guizhou Provincial Key Laboratory of Biodiversity Conservation and Utilization in the Fanjing Mountain Region, Tongren University, Tongren 554300, Guizhou, China Tongren University Tongren China; 2 Central South Inventory and Planning Institute of National Forestry and Grassland Administration, Changsha, Hunan 410014, China Central South Inventory and Planning Institute of National Forestry and Grassland Administration Changsha China

**Keywords:** Arachnida, biodiversity, diagnosis, morphology, new combination, taxonomy

## Abstract

Four new species of *Araneus* Clerck, 1757 from southern China are described: *A.mayanghe* Mi & Wang, **sp. nov.** (♂♀) from Guizhou, *A.shiwandashan* Mi & Wang, **sp. nov.** (♂♀) from Guangxi, and *A.zhoui* Mi & Wang, **sp. nov.** (♂♀) from Hainan are assigned to the *A.sturmi* group, and *A.fenzhi* Mi & Wang, **sp. nov.** (♂♀) from Hunan, Guizhou and Jiangxi is not assigned to any species group. A new combination is also proposed: *Aoaraneusoctumaculalus* (Han & Zhu, 2010) **comb. nov.**

## ﻿Introduction

Araneidae is the third most speciose family in the order Araneae, and 3119 species in 188 genera are known worldwide, of which 437 species in 54 genera have been recorded from China (WSC 2023). Data on the family in China is far from complete. For example, ongoing surveys of Araneidae from Xishuangbanna Tropical Botanical Garden in Yunnan Province, southwestern China has produced more than 120 species and about two-fifths are new to science (Mi and Li 2021a, b, 2022).

As the largest genus of the family, *Araneus* Clerck, 1757 includes 555 species worldwide (WSC 2023), and for centuries it has been a dumping ground for araneid species, making it polyphyletic, as shown by the multi-gene phylogenetic analysis of Scharff et al. (2020).

A comprehensive study of Chinese *Araneus* species was conducted by Yin et al. (1997), and a total of 79 *Araneus* species were described and illustrated. Fifteen Chinese *Araneus* species published before 1997 were not included: *A.anjonensis* Schenkel, 1963, *A.basalteus* Schenkel, 1936, *A.fengshanensis* Zhu & Song, 1994, *A.haruspex* (O. Pickard-Cambridge, 1885), *A.loczyanus* (Lendl, 1898), *A.mangarevoides* (Bösenberg & Strand, 1906), *A.metellus* (Strand, 1907), *A.paitaensis* Schenkel, 1953, *A.pecuensis* (Karsch, 1881), *A.pseudoconicus* Schenkel, 1936, *A.roseomaculatus* Ono, 1992, *A.scutellatus* Schenkel, 1963, *A.transversivittiger* (Strand, 1907), *A.triangulus* (Fox, 1938), and *A.zygielloides* Schenkel, 1963. Since then, 35 new species and a new record of the genus *Araneus* have been reported (Zhu et al. 1998, 2005; Hu 2001; Zhang and Zhang 2002; Zhang et al. 2006; Yin et al. 2007, 2009; Han and Zhu 2010; Guo et al. 2011; Zhou et al. 2017; Liu et al. 2019, 2022; Mi and Li 2022). To date, including generic changes of 11 species (*A.acusisetus* Zhu & Song, 1994, *A.baotianmanensis* Hu, Wang & Wang, 1991, *A.henanensis* (Hu, Wang & Wang, 1991), *A.himalayaensis* Tikader, 1975, *A.inustus* (L. Koch, 1871), *A.mitificus* (Simon, 1886), *A.nigromaculatus* Schenkel, 1963, *A.nympha* (Simon, 1889), *A.pentagrammicus* (Karsch, 1879), *A.pineus* Yin, Wang, Xie & Peng, 1990, and *A.pseudocentrodes* Bösenberg & Strand, 1906 were transferred from *Araneus* to other genera), a total of 118 *Araneus* species have been recorded in China.

Yin et al. (1997) placed 69 *Araneus* species into six species groups (Table 1). The following 10 species were not assigned to any of the above groups because original descriptions were too simple and/or illustrations not clear enough: *A.badiofoliatus* Schenkel, 1963, *A.badongensis* Song & Zhu, 1992, *A.decentellus* Strand, 1907, *A.diffinis* Zhu, Tu & Hu, 1988, *A.doenitzellus* Strand, 1906, *A.hetian* Hu & Wu, 1989, *A.nigromaculatus*, *A.pavlovi* Schenkel, 1953, *A.pichoni* Schenkel, 1963, and *A.virgus* (Fox, 1938).

**Table 1.** Yin et al.’s (1997) grouping of 69 *Araneus* species.

Species group	Included species
*A.dehaani* group	*A.dehaani* (Doleschall, 1859), *A.albomaculatus* Yin, Wang, Xie & Peng, 1990 and *A.shunhuangensis* Yin, Wang, Xie & Peng, 1990
*A.ejusmodi* group	*A.cercidius* Yin, Wang, Xie & Peng, 1990, *A.ejusmodi* Bösenberg & Strand, 1906, *A.elongatus* Yin, Wang & Xie, 1989, *A.inustus*, *A.mitificus*, *A.pseudocentrodes*, *A.tengxianensis* Zhu & Zhang, 1994 and *A.viridiventris* Yaginuma, 1969
*A.sturmi* group	*A.acusisetus*, *A.auriculatus* Song & Zhu, 1992, *A.circellus* Song & Zhu, 1992, *A.colubrinus* Song & Zhu, 1992, *A.dayongensis* Yin, Wang, Xie & Peng, 1990, *A.nympha*, *A.octodentalis* Song & Zhu, 1992, *A.pseudosturmii* Yin, Wang, Xie & Peng, 1990, *A.sturmi* (Hahn, 1831), *A.vermimaculatus* Zhu & Wang, 1994, *A.viperifer* Schenkel, 1963, *A.wulongensis* Song & Zhu, 1992 and *A.yuanminensis* Yin, Wang, Xie & Peng, 1990
*A.diadematus* group	*A.affinis* Zhu, Tu & Hu, 1988, *A.aksuensis* Yin, Xie & Bao, 1996, *A.ancurus* Zhu, Tu & Hu, 1988, *A.angulatus* Clerck, 1757, *A.baotianmanensis*, *A.beijiangensis* Hu & Wu, 1989, *A.bicavus* Zhu & Wang, 1994, *A.biprominens* Yin, Wang & Xie, 1989, *A.boesenbergi* (Fox, 1938), *A.chunhuaia* Zhu, Tu & Hu, 1988, *A.circumbasilaris* Yin, Wang, Xie & Peng, 1990, *A.diadematoides* Zhu, Tu & Hu, 1988, *A.diadematus* Clerck, 1757, *A.flagelliformis* Zhu & Yin, 1998, *A.flavidus* Yin, Wang, Xie & Peng, 1990, *A.gratiolus* Yin, Wang, Xie & Peng, 1990, *A.guandishanensis* Zhu, Tu & Hu, 1988, *A.himalayaensis*, *A.licenti* Schenkel, 1953, *A.linshuensis* Yin, Wang, Xie & Peng, 1990, *A.marmoreus* Clerck, 1757, *A.marmoroides* Schenkel, 1953, *A.motuoensis* Yin, Wang, Xie & Peng, 1990, *A.nidus* Yin & Gong, 1996, *A.pahalgaonensis* Tikader & Bal, 1981, *A.pentagrammicus*, *A.pinguis* (Karsch, 1879), *A.taigunensis* Zhu, Tu & Hu, 1988, *A.tetraspinulus* (Yin, Wang, Xie & Peng, 1990), *A.tubabdominus* Zhu & Zhang, 1993, *A.tsuno* Yaginuma, 1972, *A.xianfengensis* Song & Zhu, 1992 and *A.zebrinus* Zhu & Wang, 1994
*A.vermimaculatus* group	*A.menglunensis* (Yin, Wang, Xie & Peng, 1990), *A.miquanensis* Yin, Wang, Xie & Peng, 1990, *A.pineus*, *A.pseudoventricosus*, *A.tartaricus* (Kroneberg, 1875), *A.tenerius* Yin, Wang, Xie & Peng, 1990, *A.tricoloratus* Zhu, Tu & Hu, 1988, *A.variegatus* Yaginuma, 1960, *A.ventricosus* (L. Koch, 1878) and *A.yuzhongensis* Yin, Wang, Xie & Peng, 1990
*A.henanensis* group	*A.henanensis* and *A.yunnanensis* Yin, Peng & Wang, 1994

While examining the Araneidae specimens collected in several national nature reserves in southern China, four new species of *Araneus* are identified, and they are described in this paper.

## ﻿Materials and methods

All specimens were collected by beating shrubs or by hand and are preserved in 75% ethanol. Type specimens of the new species are deposited in the Museum of Tongren University, China (**TRU**). The specimens were examined with an Olympus SZX16 stereomicroscope. The epigynes were cleared in lactic acid for examination and imaging. The left male pedipalp was dissected in ethanol for examination, description, and imaging. Photographs of the habitus and copulatory organs were taken with a Kuy Nice digital camera mounted on an Olympus BX43 compound microscope. Compound focus images were generated using Helicon Focus v. 6.7.1. The paths of the left copulatory ducts were drawn using Adobe Illustrator CC 2018.

All measurements are given in millimeters. Leg measurements are given as total length (femur, patella + tibia, metatarsus, tarsus). Abbreviations used in the text and figures are as follows: **ALE** anterior lateral eye; **AME** anterior median eye; **C** conductor; **CD** copulatory duct; **CO** copulatory opening; **E** embolus; **EL** embolic lamella; **ET** embolic tooth; **FD** fertilization duct; **MA** median apophysis; **MOA** median ocular area; **PLE** posterior lateral eye; **PME** posterior median eye; **Sc** scape; **Sp** spermatheca; **ST** subterminal apophysis; **TA** terminal apophysis.

## ﻿Taxonomy

### ﻿Family Araneidae Clerck, 1757

#### ﻿Genus Araneus

Taxon classificationAnimaliaAraneaeAraneidae

Clerck, 1757

C4565E0F-A6D0-58D4-B650-71402D95D4FC

Araneus Clerck, 1757: 22.

##### Type species.

*Araneusangulatus* Clerck, 1757.

##### Comments.

Although the four new species are different from the generotype *A.angulatus* in thier smaller body size and having the abdomen lacking a pair of anterolateral humps, the epigynal base not elongated, and a shorter scape which does not extend far beyond the epigastric furrow, we provisionally place them in this genus because they share a very similar habitus and copulatory organs with other *Araneus* species. There is no doubt that their generic position needs confirmation, but it is not discussed here due to limited evidence.

#### ﻿ Araneus fenzhi

Taxon classificationAnimaliaAraneaeAraneidae

Mi & Wang sp. nov.

FCB0D573-1845-5EBB-8FBE-D8AC488E1324

https://zoobank.org/4F58C4BE-5F33-44A6-BD75-3233B75D7E48

**Figure 1.**
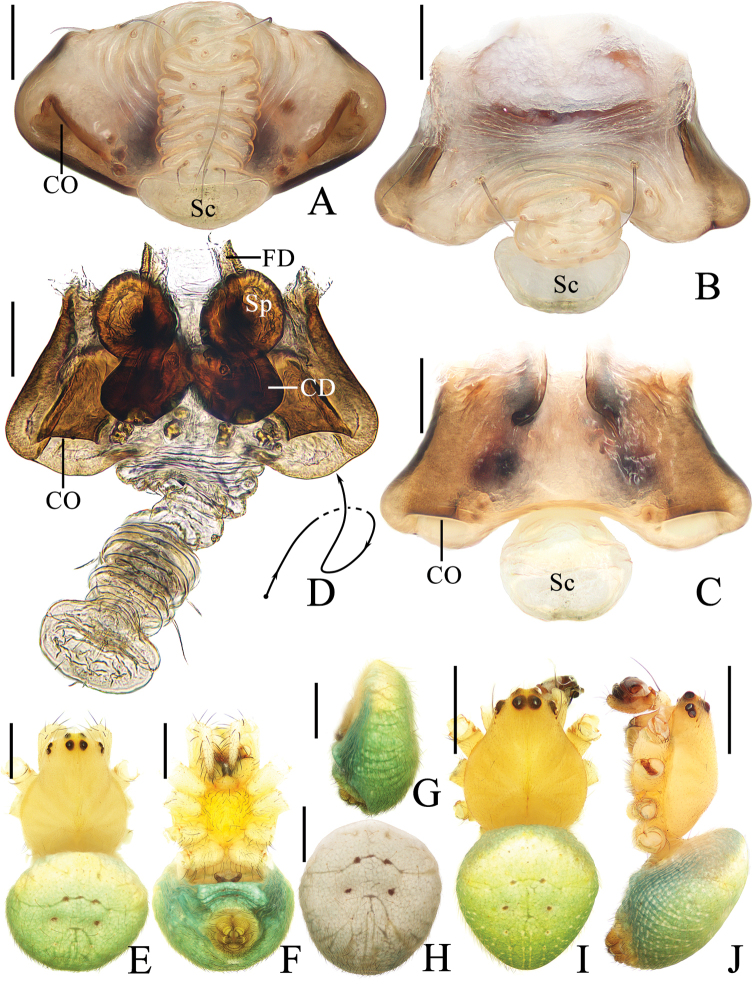
*Araneusfenzhi* sp. nov. **A–H** female paratype TRU-Araneidae-137 **I, J** male holotype **A** epigyne, ventral view **B** ibid., anterior view **C** ibid., posterior view **D** vulva, posterior view **E** habitus, dorsal view **F** ibid., ventral view **G** abdomen, lateral view **H** ibid., dorsal view **I** habitus, dorsal view **J** ibid., lateral view. Scale bars: 0.1 mm (**A–D**); 1 mm (**E–J**).

**Figure 2.**
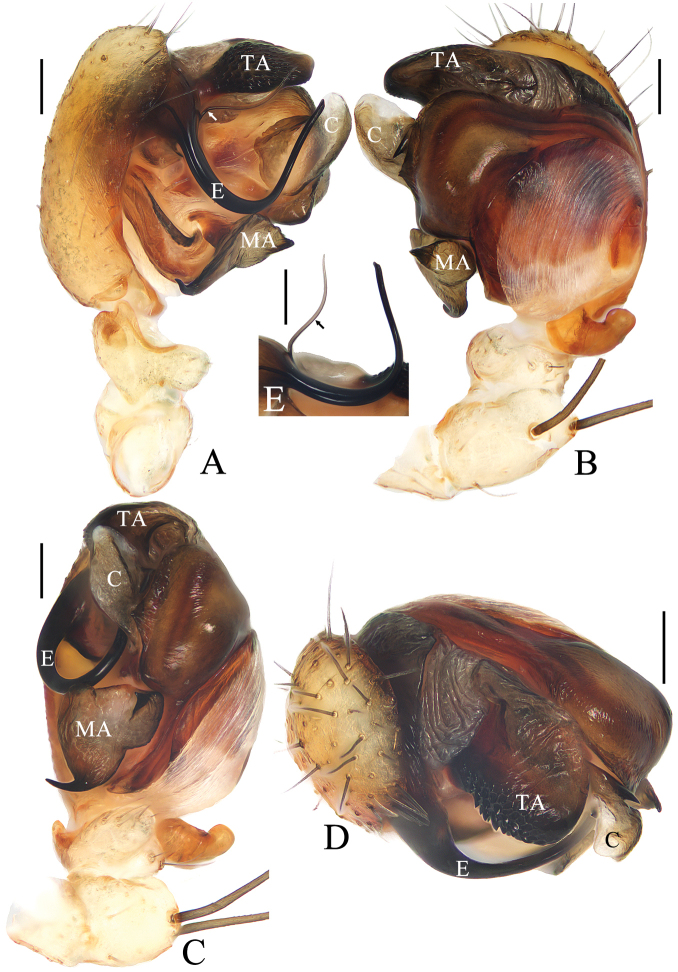
*Araneusfenzhi* sp. nov. **A, E** male paratype TRU-Araneidae-138 **B–D** male holotype **A** pedipalp, prolateral view **B** ibid., retrolateral view **C** ibid., ventral view **D** ibid., apical view **E** embolus. Scale bars: 0.1 mm.

Figs 1 , 2 , 9A–D , 10

##### Type material.

***Holotype*** ♂ (TRU-Araneidae-136), China: Hunan Province, Shaoyang City, Suining County, Zhaishi Township, Huangsang National Nature Reserve (26°23.51'N, 110°9.56'E, ca 1620 m), 11.VII.2022, X.Q. Mi & C. Wang leg. ***Paratypes***: 1♂2♀ (TRU-Araneidae-137–139), same data as for holotype; 2♂4♀ (TRU-Araneidae-140–145), Guizhou Province, Qiandongnan Miao and Dong Autonomous Prefecture, Leishan County, Danjiang Township, Leigongshan National Nature Reserve (26°22.99'N, 108°12.08'E, ca 1990 m), 20.VII.2017, C. Wang et al. leg.; 1♂ (TRU-Araneidae-146), Jiangxi Province, Ji’an City, Anfu County, Taishan Township, Wugongshan (27°27.50'N, 114°10.08'E, ca 1270 m), 23.VI.2022, Z.G. Huang et al. leg.

##### Etymology.

The specific name is derived from the Chinese pinyin “fenzhi”, meaning branched, referring to the threadlike branch of the embolus.

##### Diagnosis.

The new species resembles *A.albabdominalis* Zhu, Zhang, Zhang & Chen, 2005 in having pale green coloration in life and a similar shape of the median apophysis and conductor, but it can be distinguished as follows: 1) epigyne scape not twisted into an S-shape vs S-shaped (Zhu et al. 2005: fig. 3B); 2) copulatory openings wedge-shaped in ventral view vs round (Zhu et al. 2005: fig. 3B); 3) embolus of male pedipalp U-shaped in prolateral view vs straight (Zhu et al. 2005: fig. 3D); 4) the branch on the embolus is threadlike vs lamellar (Zhu et al. 2005: fig. 3D); and 5) carapace unicolor vs with a pair of lateral longitudinal patches (Zhu et al. 2005: fig. 3A).

##### Description.

**Male** (holotype, Figs 1I, J, 2B–D, 9A–D; paratype TRU-Araneidae-138, Fig. 2A, E). Total length 3.35. Carapace 1.75 long, 1.20 wide. Abdomen 2.10 long, 1.65 wide. Clypeus 0.10 high. Eye sizes and interdistances: AME 0.10, ALE 0.06, PME 0.11, PLE 0.06, AME–AME 0.15, AME–ALE 0.15, PME–PME 0.13, PME–PLE 0.20, MOA length 0.33, anterior width 0.35, posterior width 0.33. Leg measurements: I 6.90 (2.05, 2.40, 1.70, 0.75), II 6.45 (1.95, 2.20, 1.60, 0.70), III 3.60 (1.20, 1.10, 0.80, 0.50), IV 5.25 (1.70, 1.70, 1.30, 0.55). Carapace pear-shaped, yellow; base of eyes brown. Cervical groove inconspicuous; fovea longitudinal. Chelicerae yellow; four promarginal and three retromarginal teeth. Endites almost square, yellow with very narrow, dark anterior edge, with tooth-like process laterally. Labium triangular, yellow. Sternum cordiform, yellow with dark setae. Legs yellow to brown, without annuli; femur I with eight macrosetae; tibia I with 10 macrosetae; tibia II with 12 macrosetae; tibia III with nine macrosetae; tibia IV with seven macrosetae. Abdomen oval, ~1.3× longer than wide, yellowish green with pale yellow spots; venter whitish green. Spinnerets yellowish brown.

***Pedipalp*** (Fig. 2) with a basal femoral protrusion; patella with two bristles; median apophysis almost triangular, with a single long, slender, curved spur and a shorter broad spur; embolus twisted into a U-shape, with a threadlike branch at base, without cap (see arrows in Fig. 2A, E); conductor membranous, with a spur at base; terminal apophysis blunt, with dense denticles.

**Female** (paratype TRU-Araneidae-137, Fig. 1A–H). Total length 4.40. Carapace 2.35 long, 1.85 wide. Abdomen 2.80 long, 2.55 wide. Clypeus 0.10 high. Eye sizes and interdistances: AME 0.11, ALE 0.08, PME 0.13, PLE 0.08, AME–AME 0.18, AME–ALE 0.25, PME–PME 0.15, PME–PLE 0.33, MOA length 0.40, anterior width 0.40, posterior width 0.38. Leg measurements: I 8.30 (2.45, 2.95, 2.05, 0.85), II 7.70 (2.25, 2.70, 1.95, 0.80), III 4.75 (1.55, 1.50, 1.05, 0.65), IV 6.85 (2.25, 2.30, 1.60, 0.70). Habitus similar to that of male, but abdomen ~1.1× wider than long.

**Figure 3.**
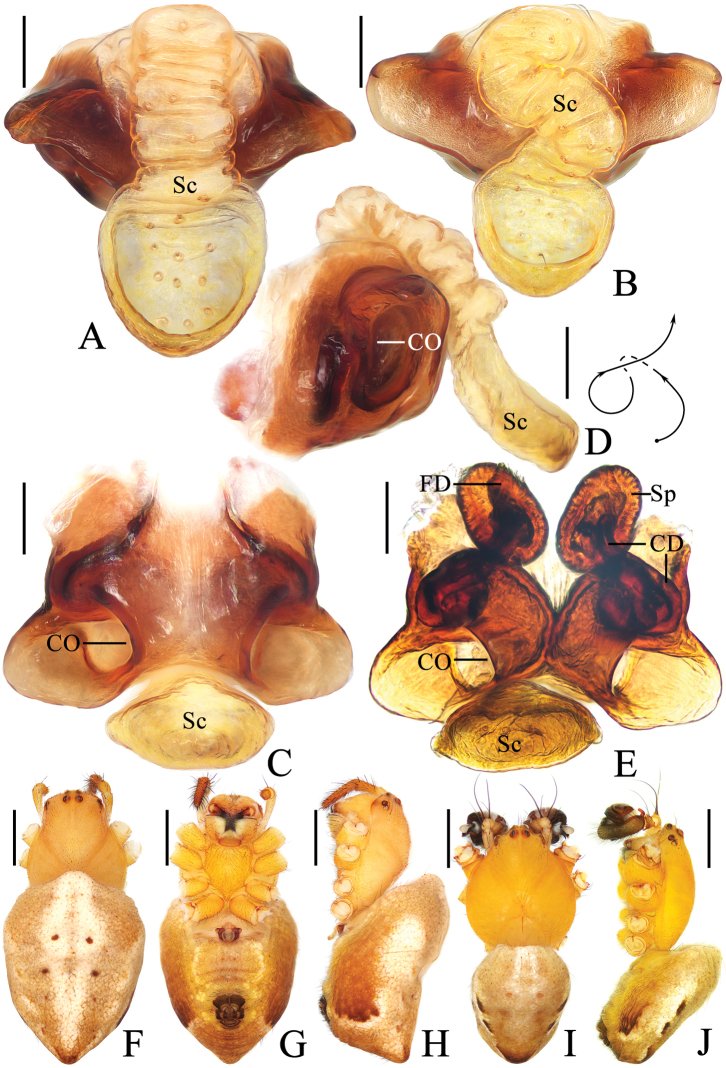
*Araneusmayanghe* sp. nov. **A, C–H** female paratype TRU-Araneidae-149 **B** female paratype TRU-Araneidae-150 **I, J** male holotype **A** epigyne, ventral view **B** ibid., ventral view **C** ibid., posterior view dorsal view **D** ibid., lateral view **E** vulva, posterior view **F** habitus, dorsal view **G** ibid., ventral view **H** ibid., lateral view habitus **I** ibid., dorsal view **J** ibid., lateral view. Scale bars: 0.1 mm (**A–E**); 1 mm (**F–J**).

***Epigyne*** (Fig. 1A–D) ~1.65× wider than long; scape with nearly parallel sides, spoon shaped distally; copulatory openings wedge shaped in ventral view, near posterior margin; copulatory ducts coiled ~360°, ~2× longer than a spermatheca diameter; spermathecae spherical, ~1/4 spermatheca diameter apart.

##### Variation.

Total length: ♂♂ 2.95–3.40 (*n* = 5); ♀♀ 4.15–5.65 (*n* = 6). Preserved specimens grayish white. The embolic branch is generally broken off.

##### Distribution.

Hunan, Guizhou, Jiangxi.

##### Comment.

The oval abdomen is similar to that of members of the *A.ejusmodi* group, but the long, distally spoon-shaped scape differs greatly than in those species, and thus the group to which the new species belongs is unclear.

#### ﻿ Araneus mayanghe

Taxon classificationAnimaliaAraneaeAraneidae

Mi & Wang sp. nov.

1CCAAAB4-668C-5875-BD69-C772C0E2002A

https://zoobank.org/0151AD54-FB49-424C-980D-21E4D80229DA

**Figure 4.**
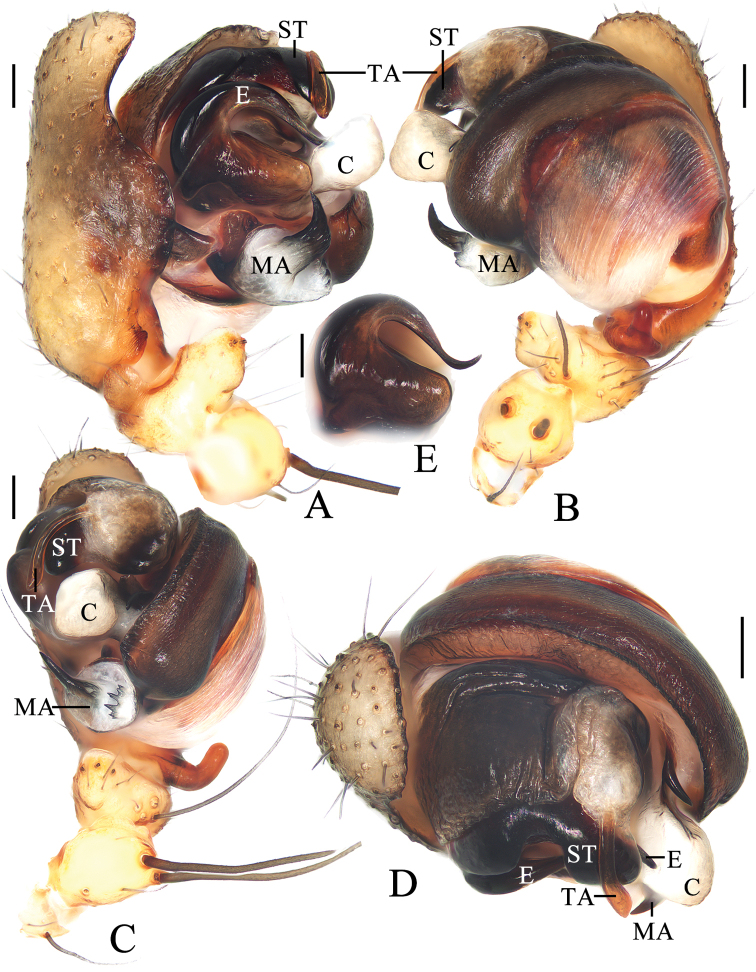
*Araneusmayanghe* sp. nov. **A–D** male holotype **E** male paratype TRU-Araneidae-148 **A** pedipalp, prolateral view **B** ibid., retrolateral view **C** ibid., ventral view **D** ibid., apical view. **E** embolus, prolateral view. Scale bars: 0.1 mm.

Figs 3 , 4 , 9E–H , 10

##### Type material.

***Holotype*** ♂ (TRU-Araneidae-147), China: Guizhou Province, Tongren City, Yanhe Tujia Autonomous County, Siqu Township, Dabao Village, Mayanghe National Nature Reserve (28°39.48'N, 108°12.80'E, ca 690 m), 19.IV.2022, X.Q. Mi et al. leg. ***Paratypes***: 4♂10♀ (TRU-Araneidae-148–161), same data as for holotype.

##### Etymology.

The specific name is a noun in apposition and refers to the type locality.

##### Diagnosis.

The new species resembles *A.ryukyuanus* Tanikawa, 2001, *A.tsurusakii* Tanikawa, 2001, *A.polydentatus*Yin et al., 2007, and *A.yuanminensis* Yin, Wang, Xie & Peng, 1990 in appearance, but differs from *A.ryukyuanus* in having 1) the copulatory openings located on the posterior surface vs the ventral surface (Tanikawa 2001: fig. 20); 2) the embolus long, reaching the base of the conductor vs short and not reaching the conductor (Tanikawa 2001: fig. 21); and 3) the embolus somewhat S-shaped distally vs C-shaped (Tanikawa 2001: fig. 21). It can be distinguished from *A.tsurusakii* in having 1) the epigyne scape almost straight or slightly twisted vs extremely twisted (Tanikawa 2001: fig. 14); 2) the copulatory opening about ~0.8× the median plate width vs ~0.33× the median plate width (Tanikawa 2001: fig. 15); and 3) the embolus somewhat S-shaped distally vs C-shaped (Tanikawa 2001: figs 16–18). It differs from *A.polydentatus* in having 1) the epigyne scape almost straight vs S-shaped (Yin et al. 2007: fig. 1g); 2) the embolus S-shaped distally vs straight (Yin et al. 2007: fig. 1b); and 3) the embolus long, reaching the base of the conductor vs short and not reaching the conductor (Yin et al. 2007: fig. 1b). It differs from *A.yuanminensis* in having 1) the copulatory openings located on the posterior surface vs on the ventral surface (Yin et al. 1990: figs 66–68); 2) the spermathecae oval vs spherical (Yin et al. 1990: fig. 68); and 3) the spinnerets at the posterior 1/3 of abdomen vs at the middle of abdomen (Yin et al. 1990: fig. 65).

##### Description.

**Male** (holotype, Figs 3I, J, 4A–D, 9E–H; paratype TRU-Araneidae-148, Fig. 4E). Total length 4.00. Carapace 2.05 long, 1.80 wide. Abdomen 2.45 long, 1.65 wide. Clypeus 0.10 high. Eye sizes and interdistances: AME 0.08, ALE 0.08, PME 0.11, PLE 0.08, AME–AME 0.15, AME–ALE 0.23, PME–PME 0.10, PME–PLE 0.28, MOA length 0.30, anterior width 0.33, posterior width 0.33. Leg measurements: I 7.30 (2.30, 2.65, 1.60, 0.75), II 7.05 (2.15, 2.65, 1.55, 0.70), III 3.75 (1.10, 1.35, 0.80, 0.50), IV 5.35 (1.80, 2.00, 1.00, 0.55). Carapace pear-shaped, yellow with dark setae; posterior eyes with brown base. Cervical groove obvious; fovea longitudinal. Chelicerae yellow; four promarginal and three retromarginal teeth. Endites yellow with very narrow, dark anterior edge, with tooth-like process laterally. Labium triangular, yellow. Sternum cordiform, yellow with dark setae. Legs yellow to dark brown, without annuli; femur I with seven macrosetae; tibia I with 11 macrosetae; tibia II with 11 macrosetae; tibia III with 10 macrosetae, tibia IV with 11 macrosetae. Abdomen oval, blunt anteriorly and pointed posteriorly, ~1.5× longer than wide, covered with gray setae; dorsum yellow with a large terminal dark patch and two pairs of lateral dark patches; venter grayish yellow. Spinnerets dark brown.

***Pedipalp*** (Fig. 4) with basal femoral protrusion; patella with two bristles; median apophysis large, bifurcated, dorsal ramus long, curved, pointed at tip; ventral ramus short with serrated tip; embolus stout at base, slender and twisted into an S-shape distally, without cap; conductor subquadrate in retrolateral view, thickened at tip, with spur at base; terminal apophysis weakly sclerotized, digitiform; subterminal apophysis large, heavily sclerotized, concave medially.

**Female** (paratype TRU-Araneidae-149, Fig. 3A, C–H; paratype TRU-Araneidae-150, Fig. 3B). Total length 4.95. Carapace 2.25 long, 1.60 wide. Abdomen 3.45 long, 2.65 wide. Clypeus 0.08 high. Eye sizes and interdistances: AME 0.09, ALE 0.09, PME 0.13, PLE 0.09, AME–AME 0.15, AME–ALE 0.30, PME–PME 0.15, PME–PLE 0.33, MOA length 0.33, anterior width 0.30, posterior width 0.35. Leg measurements: I 7.70 (2.40, 2.90, 1.65, 0.75), II 6.85 (2.10, 2.60, 1.45, 0.70), III 4.15 (1.35, 1.45, 0.85, 0.50), IV 6.10 (2.00, 2.20, 1.35, 0.55). Habitus similar to that of male but pointed anteriorly and abdominal patches much paler.

***Epigyne*** (Fig. 3A, C–E) scape almost straight, distally spoon-shaped; copulatory openings on posterior surface; copulatory ducts twisted, longer than spermatheca; spermathecae oval, not touching.

##### Variation.

Total length: ♂♂ 3.55–4.00 (*n* = 5); ♀♀ 4.15–5.55 (*n* = 10). Scape of female paratype TRU-Araneidae-149 slightly twisted into an S-shape (Fig. 3B). Abdomen of male paratype TRU-Araneidae-151 dorsally with a large, grayish brown, triangular patch.

##### Distribution.

Known only from the type locality (Guizhou).

##### Comment.

The oval, posteriorly pointed abdomen and the long, distally spoon-shaped scape indicate that the new species belongs to the *A.sturmi* group. Based on the similarities of the somatic morphology and genitalia, the new species is most similar to *A.polydentatus*, *A.ryukyuanus*, *A.tsurusakii*, *A.viperifer*, and *A.yuanminensis*.

#### ﻿ Araneus shiwandashan

Taxon classificationAnimaliaAraneaeAraneidae

Mi & Wang sp. nov.

5047ABBB-D895-51F8-848B-770F59BCE83E

https://zoobank.org/DF107666-FB11-4133-BFA7-8FED554A5C99

**Figure 5.**
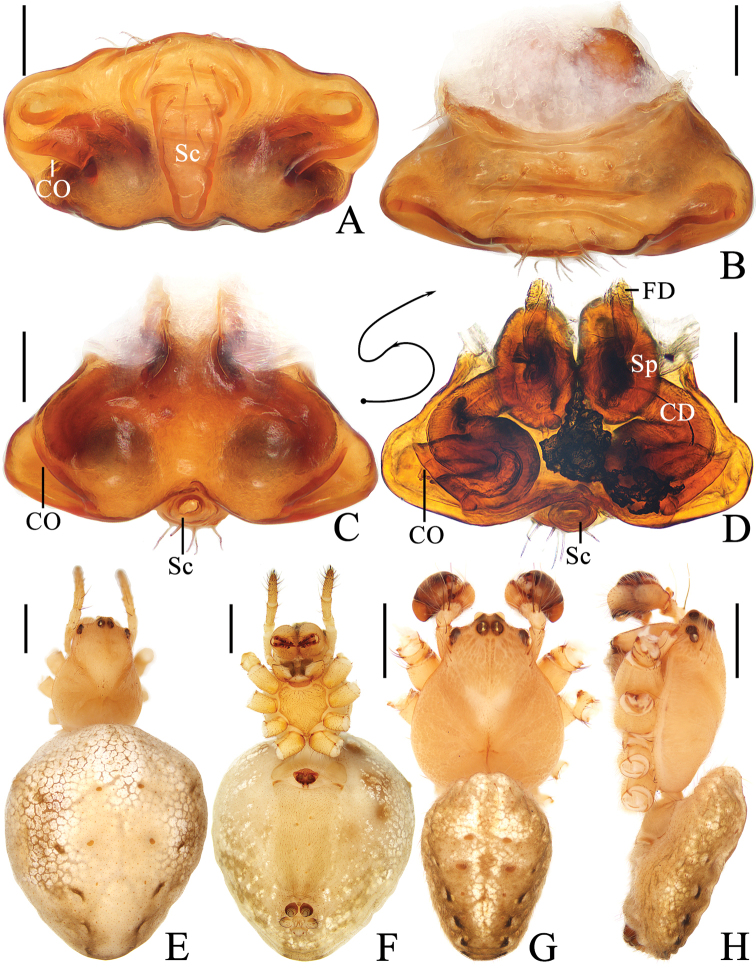
*Araneusshiwandashan* sp. nov. **A–F** female paratype TRU-Araneidae-163 **G, H** male holotype **A** epigyne, ventral view **B** ibid., anterior view **C** ibid., posterior view **D** vulva, posterior view **E** habitus, dorsal view **F** ibid., ventral view **G** ibid., dorsal view **H** ibid., lateral view. Scale bars: 0.1 mm (**A–D**); 1 mm (**E–H**).

**Figure 6.**
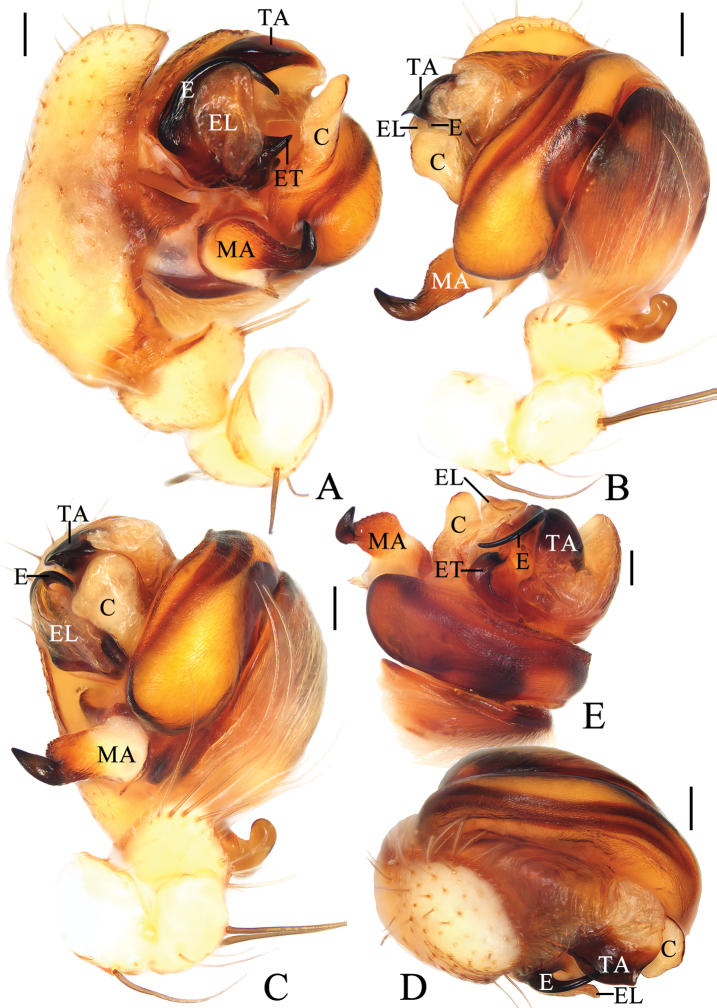
*Araneusshiwandashan* sp. nov. male holotype **A** pedipalp, prolateral view **B** ibid., retrolateral view **C** ibid., ventral view **D** ibid., apical view **E** expanded bulb, retrolateral view. Scale bars: 0.1 mm.

Figs 5 , 6 , 9I–L , 10

##### Type material.

***Holotype*** ♂ (TRU-Araneidae-162), China: Guangxi Zhuang Autonomous Region, Fangchenggang City, Shangsi County, Shiwandashan National Nature Reserve (21°52.97'N, 107°54.88'E, ca 720 m), 6.X.2018, X.Q. Mi et al. leg. ***Paratype***: 1♀ (TRU-Araneidae-163), same data as for holotype.

##### Etymology.

The specific name is a noun in apposition and refers to the type locality.

##### Diagnosis.

The new species resembles *Araneusfloriformis* Liu, Li, Mi & Peng, 2022 in somatic morphology and pedipalp structures, but differs in having 1) the median apophysis bifurcated vs uniramous (Liu et al. 2022: fig. 4A–C); 2) the embolic lamella covering the embolus medially in prolateral view vs not covering the embolus medially (Liu et al. 2022: fig. 4A, D); 3) copulatory openings at the posterior surface vs laterally (Liu et al. 2022: fig. 4G, H); and 4) the epigyne scape triangular vs spoon-shaped (Liu et al. 2022: fig. 4G, H).

##### Description.

**Male** (holotype, Figs 5G, H, 6, 9I–L). Total length 4.70. Carapace 2.50 long, 2.05 wide. Abdomen 2.80 long, 1.75 wide. Clypeus 0.13 high. Eye sizes and interdistances: AME 0.15, ALE 0.13, PME 0.15, PLE 0.13, AME–AME 0.15, AME–ALE 0.28, PME–PME 0.08, PME–PLE 0.33, MOA length 0.38, anterior width 0.38, posterior width 0.35. Leg measurements: I 9.35 (2.80, 3.40, 2.30, 0.85), II 8.60 (2.60, 3.10, 2.10, 0.80), III 4.90 (1.60, 1.65, 1.05, 0.60), IV 6.95 (2.15, 2.40, 1.70, 0.70). Carapace pear-shaped, yellow, with dark setae on sides of cephalic region. Cervical groove inconspicuous; fovea longitudinal. Chelicerae yellow; four promarginal and three retromarginal teeth. Endites yellow with very narrow, dark anterior edge, with tooth-like process laterally. Labium triangular, yellow, paler at tip. Sternum cordiform, yellow with dark setae. Legs yellow with indistinct, yellowish-brown annuli; femur I with seven macrosetae; tibia I with 10 macrosetae; tibia II with 12 macrosetae, tibia III with eight macrosetae; and tibia IV with 11 macrosetae. Abdomen oval, ~1.6× longer than wide, covered with pale setae; dorsum whitish yellow with six pairs of lateral patches; posterior patches darker; venter grayish yellow. Spinnerets brown.

***Pedipalp*** (Fig. 6) with basal femoral protrusion; patella with two bristles; median apophysis prominent, bifurcate, dorsal ramus long, curved, ventral ramus short, straight; embolus strongly sclerotized, with basal tooth, distally semicircular, embolic lamella long, covering part of embolus; conductor membranous, subquadrate in ventral view; terminal apophysis flattened, heavily sclerotized distally.

**Female** (paratype TRU-Araneidae-163, Fig. 5A–F). Total length 7.25. Carapace 2.45 long, 2.00 wide. Abdomen 5.10 long, 4.55 wide. Clypeus 0.08 high. Eye sizes and interdistances: AME 0.15, ALE 0.13, PME 0.15, PLE 0.13, AME–AME 0.15, AME–ALE 0.33, PME–PME 0.10, PME–PLE 0.40, MOA length 0.35, anterior width 0.38, posterior width 0.33. Leg measurements: I 7.85 (2.30, 2.85, 1.90, 0.80), II 7.10 (2.15, 2.50, 1.70, 0.75), III 4.30 (1.45, 1.40, 0.90, 0.55), IV 6.35 (2.10, 2.10, 1.50, 0.65). Femur I with a distinct macroseta prolaterally, tibia I with three distinct macrosetae prolaterally. Habitus similar to that of male.

***Epigyne*** (Fig. 5A–D) ~1.9× wider than long; scape triangular, ~1.8× longer than wide, directed posteriorly; copulatory openings concave, located at posterolaterally; copulatory ducts twisted into an S-shape, longer than spermatheca; spermathecae elliptical, touching each other.

##### Distribution.

Known only from the type locality (Guangxi).

##### Comments.

The oval abdomen and the male pedipalp with a bifurcated median apophysis and arched terminal apophysis indicate that the new species belongs to the *A.sturmi* group, although the scape is not twisted or widened distally. Based on the somatic morphology and genitalia, the new species is most similar to *A.breviscapus*, *A.floriformis*, and *A.pianmaensis*.

#### ﻿ Araneus zhoui

Taxon classificationAnimaliaAraneaeAraneidae

Mi & Wang sp. nov.

935B8385-040C-5AB8-A616-BF3A7FD3E9D7

https://zoobank.org/C2732937-19D2-4F05-BBA9-019024985A53

**Figure 7.**
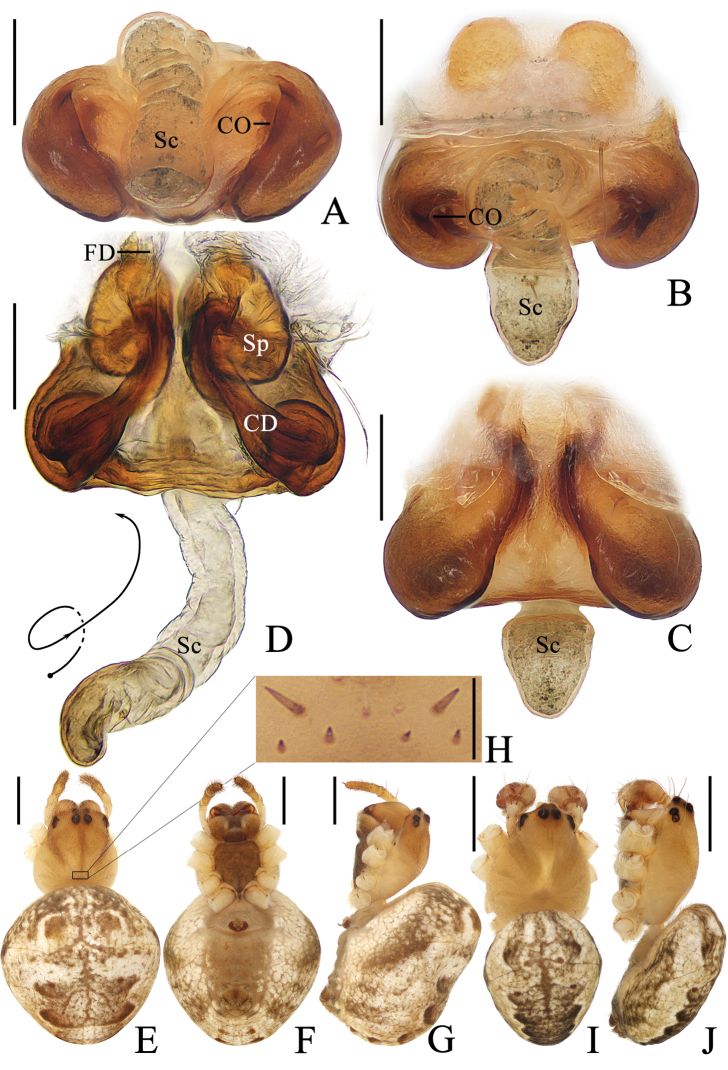
*Araneuszhoui* sp. nov. **A–H** female paratype TRU-Araneidae-165 **I, J** male holotype **A** epigyne, ventral view **B** ibid., anterior view **C** ibid., posterior view **D** vulva, posterior view **E** habitus, dorsal view **F** ibid., ventral view **G** ibid., lateral view **H** part of carapace, dorsal view **I** habitus, dorsal view **J** ibid., lateral view. Scale bars: 0.1 mm (**A–D, H**); 1 mm (**E–G, I, J**).

**Figure 8.**
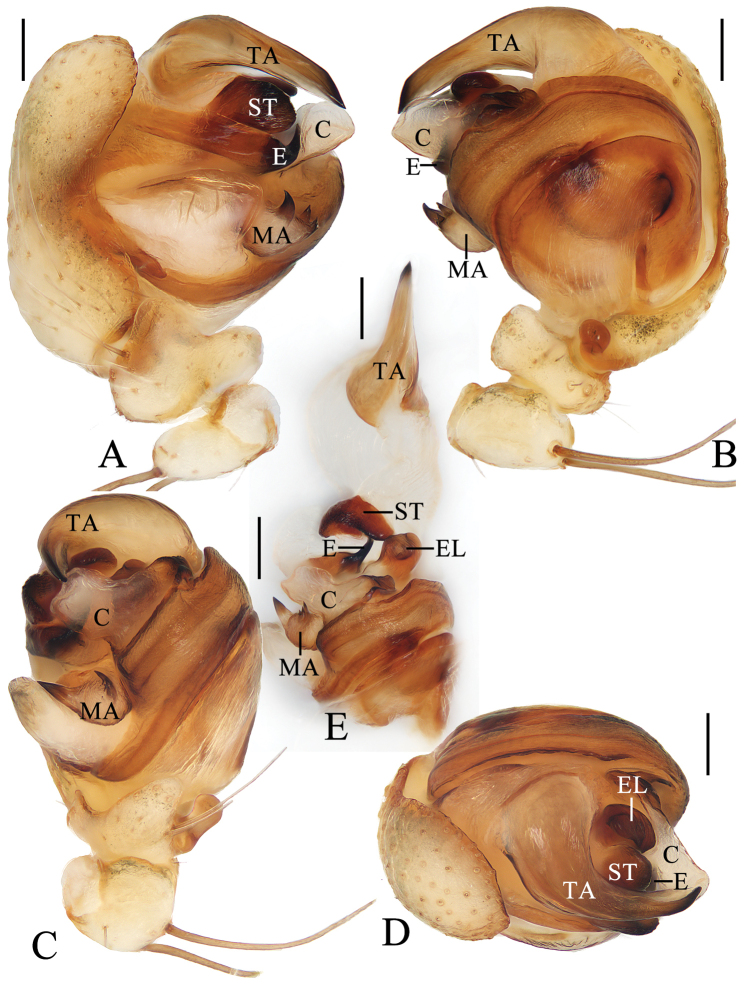
*Araneuszhoui* sp. nov. male holotype **A** pedipalp, prolateral view **B** ibid., retrolateral view **C** ibid., ventral view **D** ibid., apical view **E** expanded bulb, ventral view. Scale bars: 0.1 mm.

**Figure 9.**
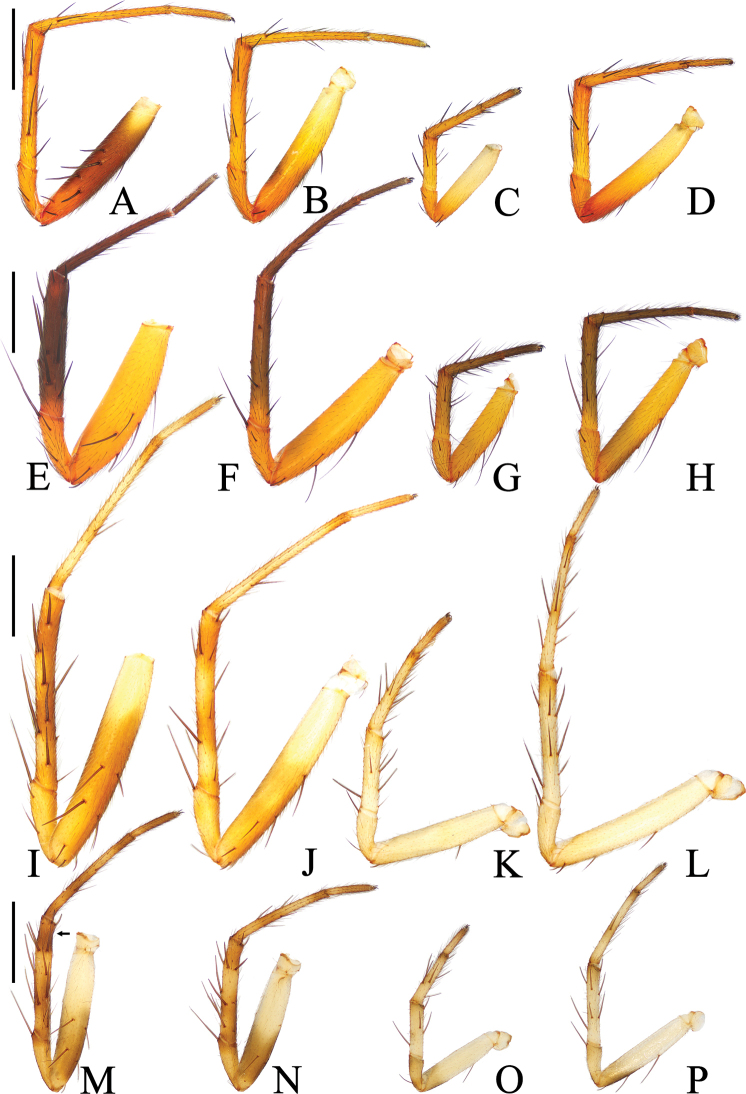
Legs of *Araneus* spp., male holotypes, prolateral view **A–D***A.fenzhi* sp. nov. **E–H***A.mayanghe* sp. nov. **I–L***A.shiwandashan* sp. nov. **M–P***A.zhoui* sp. nov. **A, E, I, M** legs I; **B, F, J, N** legs II; **C, G, K, O** legs III; **D, H, L, P** legs IV. Scale bars: 1 mm.

**Figure 10.**
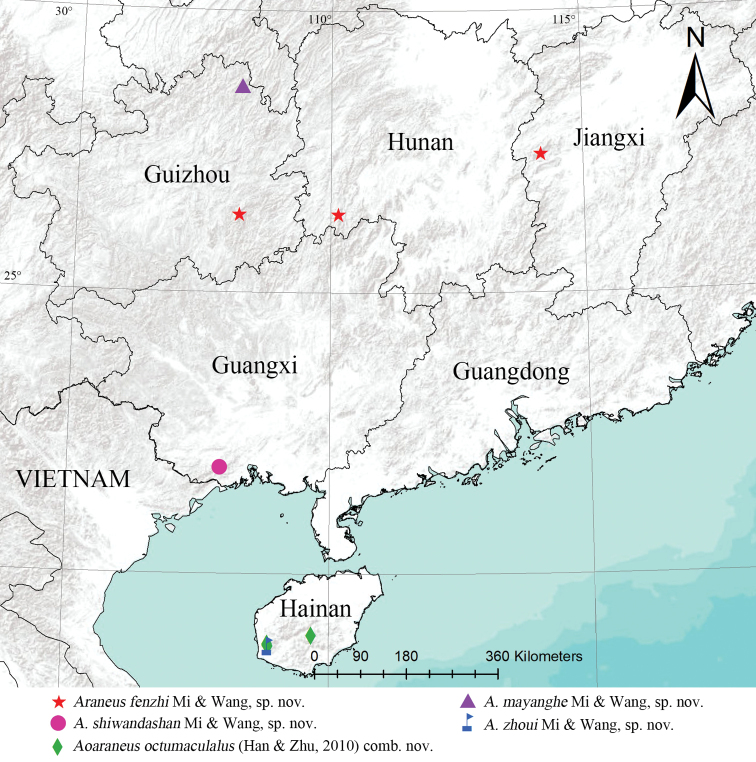
Distribution map of the species.

Figs 7 , 8 , 9M–P , 10

##### Type material.

***Holotype*** ♂ (TRU-Araneidae-164), China: Hainan Province, Ledong Li Autonomous County, Jianfeng Township, Jianfengling National Nature Reserve (18°43.11'N, 108°52.32'E, ca 1400 m), 16.IV.2019, C. Wang & Y.F. Yang leg. ***Paratypes***: 3♂6♀ (TRU-Araneidae-165–173), same data as for holotype.

##### Comparative material.

*Araneuscolubrinus* Song & Zhu, 1992, 5♀, China: Guizhou Province, Yinjiang Tujia Autonomous County, Ziwei Township, Dayuanzhi Village, Huguosi (27°54.54'N, 108°46.57'E, ca 1660 m), 9.V.2020, X.Q. Mi et al. leg.

##### Etymology.

The species is named after Mr Runbang Zhou who helped us with specimen collections; noun in genitive case.

##### Diagnosis.

The new species resembles *A.conexus* Liu, Irfan, Yang & Peng, 2019 and *A.colubrinus* Song & Zhu, 1992 in somatic morphology and genital structures, but it can be distinguished from *A.conexus* in having 1) the female carapace with two short spines anterior to the fovea vs lacking (Liu et al. 2019: fig. 1D); 2) the anterior abdomen slightly elevated in lateral view vs extremely elevated to a pointed tip (Liu et al. 2019: fig. 1E); 3) the terminal apophysis long, touching the conductor in prolateral view vs short and not touching the conductor (Liu et al. 2019: fig. 2A, B, D, E); and 4) the distal end of the subterminal apophysis rounded in prolateral view vs concave (Liu et al. 2019: fig. 2A, D). It differs from *A.colubrinus* in having 1) the epigyne scape almost straight vs S-shaped and twisted (Song and Zhu 1992: fig. 4); and 2) the spermathecae touching each other vs separated by ~1.6× of the spermatheca width.

##### Description.

**Male** (holotype, Figs 7I, J, 8, 9M–P). Total length 3.20. Carapace 1.60 long, 1.40 wide. Abdomen 1.95 long, 1.55 wide. Clypeus 0.05 high. Eye sizes and interdistances: AME 0.09, ALE 0.08, PME 0.11, PLE 0.08, AME–AME 0.08, AME–ALE 0.18, PME–PME 0.08, PME–PLE 0.23, MOA length 0.28, anterior width 0.25, posterior width 0.30. Leg measurements: I 6.15 (1.85, 2.15, 1.50, 0.65), II 5.45 (1.65, 1.90, 1.30, 0.60), III 3.35 (1.10, 1.10, 0.70, 0.45), IV 4.45 (1.40, 1.50, 1.05, 0.50). Carapace pear-shaped, yellow, paler anterior to fovea; ALEs, PMEs, and PLEs with black base. Cervical groove inconspicuous; fovea depressed. Chelicerae yellow; four promarginal and three retromarginal teeth. Endites yellow with very narrow, dark anterior edge, with tooth-like process laterally. Labium triangular, yellow with paler tip. Sternum cordiform, yellow with grayish brown patches, with brown setae. Legs yellow with yellowish-brown annuli; femur I with eight macrosetae; tibia I with 14 macrosetae, distally with constriction (see arrow in Fig. 9M); tibia II with 12 macrosetae; tibia III with six macrosetae; tibia IV with nine macrosetae. Abdomen oval, ~1.25× longer than wide; dorsum whitish yellow, with large grayish brown patch extending from anterior edge to posterior end, bearing four pairs of constrictions; venter grayish brown with pair of longitudinal yellow patches laterally. Spinnerets grayish brown.

***Pedipalp*** (Fig. 8) with basal femoral protrusion; patella with two bristles; median apophysis with prolateral spur and four or five small retrolateral teeth; embolus tapered, curved counterclockwise at tip; conductor membranous, widest at base; terminal apophysis approximately equal in length to bulb diameter, distally touching conductor, subterminal apophysis with blunt tip in prolateral view.

**Female** (paratype TRU-Araneidae-165, Figs 7A–H). Total length 3.80. Carapace 1.60 long, 1.40 wide. Abdomen 2.85 long, 2.55 wide. Clypeus 0.03 high. Eye sizes and interdistances: AME 0.10, ALE 0.09, PME 0.13, PLE 0.09, AME–AME 0.15, AME–ALE 0.25, PME–PME 0.13, PME–PLE 0.30, MOA length 0.28, anterior width 0.28, posterior width 0.30. Leg measurements: I 5.10 (1.55, 1.90, 1.10, 0.55), II 4.45 (1.35, 1.65, 0.95, 0.50), III 2.90 (1.00, 0.95, 0.55, 0.40), IV 4.00 (1.25, 1.40, 0.90, 0.45). Habitus similar to that of male but anterior abdominal elevation more obvious, carapace with two short spines and four teeth anterior to fovea.

***Epigyne*** (Fig. 7A–D): ~1.7× wider than long; scape with nearly parallel sides, distally directed ventrally; copulatory openings slit-like, ventral; copulatory ducts coiled ~360°, widest at origin, longer than spermatheca; spermathecae elliptical, touching each other.

##### Variation.

Total length: ♂♂ 2.85–3.20 (*n* = 4); ♀♀ 2.95–4.20 (*n* = 6). Some female carapaces only have two short spines anterior to fovea rather than four short spines.

##### Distribution.

Known only from the type locality (Hainan).

##### Comments.

The oval abdomen and the long, twisted, distally spoon-shaped scape indicate that the new species belongs to the *A.sturmi* group. The somatic morphology and genitalia indicate that the new species is most similar to *A.colubrinus* and *A.conexus*.

#### ﻿Genus Aoaraneus

Taxon classificationAnimaliaAraneaeAraneidae

Tanikawa, Yamasaki & Petcharad, 2021

E567D05A-4942-5426-924C-EEBC88013BA8

Aoaraneus Tanikawa, Yamasaki & Petcharad, 2021: 89.

##### Type species.

*Aoaraneuspentagrammicus* (Karsch, 1879).

#### ﻿ Aoaraneus octumaculalus

Taxon classificationAnimaliaAraneaeAraneidae

(Han & Zhu, 2010) comb. nov.

37533C6E-D3FB-5230-9EA8-5CA7EB1C9FBB

Fig. 10

Araneus octumaculalus Han & Zhu, 2010: 58, figs 1–6. (Type material not examined.)

##### Material examined.

1♀ (TRU-Araneidae-174), China: Hainan Province, Ledong Li Autonomous County, Jianfeng Township, Jianfengling National Nature Reserve, around Tianchi pond (18°44.45'N, 108°51.49'E, ca 860 m), 11.IV.2019, C. Wang & Y.F. Yang leg.; 1♀ (TRU-Araneidae-175), same locality and collectors (18°45.24'N, 108°51.57'E, ca 850 m), 14.IV.2019; 1♀ (TRU-Araneidae-176), Jianfengling National Nature Reserve, around the peak of Jianfengling (18°43.11'N, 108°52.32'E, ca 1400 m), 16.IV.2019, same collectors; 1♂ (TRU-Araneidae-177), Hainan Province, Wuzhishan City, Shuiman Township, around Yatai Rainforest Hotel (18°54.37'N, 109°40.70'E, ca 750 m), 11.VIII.2020, X.Q. Mi et al. leg.

##### Description.

See Han and Zhu (2010).

##### Comments.

The type locality of *A.octumaculalus* is in Changjiang County, Hainan Province. The type specimens were not examined because this species was well described and illustrated (Han and Zhu 2010), and both male and female specimens of this species from nearby localities in Hainan were examined. The new combination is based on the following characters of *Aoaraneus*: long, wrinkled and flexible scape with bent tip; long terminal and subterminal apophyses, median apophysis with apical and basal projections; male endite with lateral tooth, male coxa I with ventral hook, and male femur II with groove.

## ﻿Discussion

The *Araneusdehaani* group of Yin et al. (1997) included three species. The extreme sexual dimorphism of the species “*A.dehaani*” (now *Parawixiadehaani*) was discussed by Scharff et al. (2020), and molecular phylogenetic analysis has indicated that this species belonged to a new genus in the Backobourkiines. Because *A.albomaculatus* also shows extreme sexual dimorphism (female ~6.2× longer than male) and the morphology is similar to “*A.dehaani*”, it is likely in the same genus. However, a third species, *A.shunhuangenesis*, does not show extreme sexual dimorphism, and the long triangular epigyne, rather flattened pedipalp bulb, single macroseta on the male pedipalp patella, and the enlarged male tibia II (Yin et. al 2007) indicate that it may belong to another genus.

Yin et al. (1997) based the *A.ejusmodi* group on the following characters: the oval or elongate-oval abdomen and the epigyne with a short scape. The characters of the male padipalp were not thoroughly considered, making the *A.ejusmodi* group somewhat arbitrary. Further taxonomic and systematic work must be conducted to clarify the aforementioned issues.

## Supplementary Material

XML Treatment for Araneus

XML Treatment for Araneus fenzhi

XML Treatment for Araneus mayanghe

XML Treatment for Araneus shiwandashan

XML Treatment for Araneus zhoui

XML Treatment for Aoaraneus

XML Treatment for Aoaraneus octumaculalus
